# Prenatal Skeletal Muscle Transcriptome Analysis Reveals Novel MicroRNA-mRNA Networks Associated with Intrauterine Growth Restriction in Pigs

**DOI:** 10.3390/cells10051007

**Published:** 2021-04-24

**Authors:** Asghar Ali, Eduard Murani, Frieder Hadlich, Xuan Liu, Klaus Wimmers, Siriluck Ponsuksili

**Affiliations:** 1 Leibniz Institute for Farm Animal Biology, Institute for Genome Biology, Wilhelm-Stahl-Allee 2, 18196 Dummerstorf, Germany; ali@fbn-dummerstorf.de (A.A.); murani@fbn-dummerstorf.de (E.M.); hadlich@fbn-dummerstorf.de (F.H.); liu.xuan@fbn-dummerstorf.de (X.L.); klaus.wimmers@uni-rostock.de (K.W.); 2 Faculty of Agricultural and Environmental Sciences, University Rostock, 18059 Rostock, Germany

**Keywords:** IUGR, fetal growth, miRNAs, skeletal muscle development, miR-210

## Abstract

Intrauterine growth restriction (IUGR) occurs in 15–20% of pig neonates and poses huge economic losses to the pig industry. IUGR piglets have reduced skeletal muscle growth, which may persist after birth. Prenatal muscle growth is regulated by complex molecular pathways that are not well understood. MicroRNAs (miRNAs) have emerged as the main regulators of vital pathways and biological processes in the body. This study was designed to identify miRNA–mRNA networks regulating prenatal skeletal muscle development in pigs. We performed an integrative miRNA–mRNA transcriptomic analysis in longissimus dorsi muscle from IUGR fetuses and appropriate for gestational age (AGA) fetuses at 63 days post conception. Our data showed that 47 miRNAs and 3257 mRNAs were significantly upregulated, and six miRNAs and 477 mRNAs were significantly downregulated in IUGR compared to AGA fetuses. Moreover, 47 upregulated miRNAs were negatively correlated and can potentially target 326 downregulated genes, whereas six downregulated miRNAs were negatively correlated and can potentially target 1291 upregulated genes. These miRNA–mRNA networks showed enrichment in biological processes and pathways critical for fetal growth, development, and metabolism. The miRNA–mRNA networks identified in this study can potentially serve as indicators of prenatal fetal growth and development as well as postnatal carcass quality.

## 1. Introduction

Pig breeding programs have been traditionally focused on increasing litter size at birth and weaning [1,2]. Increased litter size affects litter uniformity, resulting in natural intrauterine growth restriction (IUGR) in some piglets [2]. Among all domestic animal species, pigs exhibit the most severe naturally occurring IUGR [3,4]. IUGR is defined as the inability of a fetus to reach its maximum growth potential, resulting in a fetal or birth weight more than two standard deviations (SD) below the mean weight for the corresponding gestational age or mean weight of littermates [4,5]. The inability of the placenta to supply or distribute sufficient nutrients and oxygen to the growing embryos/fetuses is one of the major factors in the pathogenesis of IUGR [6]. IUGR in pigs is associated with high morbidity and mortality during perinatal and pre-weaning periods, and can also lead to long term growth retardation and poor carcass quality [4,7,8]. IUGR-affected pigs have reduced feed conversion efficiency, decreased meat percentage, and high fat content in the carcass [9,10]. Along with impaired growth and development of embryo/fetus, IUGR can affect multiple fetal tissues and organ systems [4]. A recent study has shown that IUGR in piglets affects the structure, proliferation, and differentiation of skeletal muscle fibers [11].

Skeletal muscles account for nearly 45% of total body proteins and are the most abundant tissue in the body [12]. Reduced prenatal skeletal muscle growth and postnatal skeletal muscle mass are the characteristic features of IUGR [13,14]. Birth weight of piglets is directly affected by prenatal skeletal muscle development [15] and reflects the availability and utilization of nutrients for fetal growth and development [16]. Skeletal muscle growth and mass is largely determined during the prenatal period by the number and size of muscle fibers, and the number of muscle fibers does not increase after birth [17,18,19]. The contribution of the so-called satellite cells, a subpopulation of myogenic stem cells, to postnatal muscle growth is very subtle [20,21]. During the prenatal period, myofibers are formed in two waves [22]. In the first wave, myoblasts differentiate into small multinucleated primary myofibers, followed by a second wave of large secondary myofibers formation using primary myofibers as a template [22,23]. In pigs, the primary myofibers appear at around 35 days post conception (dpc) and last by 60 dpc, and the secondary myofibers start appearing at 55 dpc and their number increases by many times by 75 dpc [19,23]. Formation of myofibers ceases and the total number of myofibers is established by 85−90 dpc [17]. The ratio of secondary to primary myofibers is higher in large compared to small pig breeds [24,25]. The third wave is postnatal, starting at around 60 days after birth, during which no new muscle fibers are generated but there is a transition between slow-oxidative and fast-glycolytic fibers [23].

Impaired skeletal muscle development during the prenatal period can have a long-lasting effect on muscle growth during the postnatal period, even after compensatory growth [26]. Therefore, a better understanding of the molecular pathways regulating prenatal skeletal muscle development is required to improve pigs’ health as well as economic efficiency of pig industry. MicroRNAs (miRNAs) have emerged as important modulators of molecular pathways and cellular processes involved in animal development [27]. MiRNAs are small non-coding RNAs that are approximately 22 nucleotides long [28]. A single miRNA can regulate the expression of multiple genes through post-transcriptional repression and epigenetic regulation, making miRNAs the major regulators of gene expression [28,29,30,31,32]. Several studies have shown the role of miRNAs and their target genes in skeletal muscle growth and development at different prenatal and postnatal stages across different breeds of pigs [23,33,34,35,36,37,38].

In a previous study, we identified differentially expressed genes at seven key stages of myogenesis (14, 21, 35, 49, 63, 77 and 91 dpc) in two pig breeds, Pietrain (Pi) and Duroc, which differ evidently in muscle mass and structure [39]. In another study, we identified differentially expressed miRNAs in longissimus dorsi muscle (LDM) of German Landrace (DL) and Pi pig breeds at two prenatal stages (63 and 91 dpc) and one postnatal stage (180 days after birth) [33]. DL and Pi breeds also differ in muscularity, and Pi pigs are leaner and have a lower fat to meat ratio compared to DL pigs [33]. We reported that miRNAs from the miR-17 family and miR-17-92 cluster were upregulated at 63 dpc and were also consistently higher in Pi pigs compared to DL pigs [33]. Predicted target genes of the differentially expressed miRNAs enriched several pathways related to muscle development [33]. Other studies have demonstrated that the miR-17 family regulates cell growth and development via the downregulation of transforming growth factor beta receptor II (*TGFBR2*) and insulin-like growth factor 1 (*IGF1*) [40,41].

More integrative studies are needed to uncover the molecular pathways involved in skeletal muscle growth and development and their role in pathophysiology of IUGR. In this study, we established an F2 population by crossing DL and Pi breeds and performed an integrative miRNA–mRNA transcriptomic analysis in LDM from IUGR and appropriate for gestational age (AGA) fetuses at 63 dpc. The aim of this study was to investigate gene networks associated with skeletal muscle development and pathophysiology of IUGR. This study provides valuable insights for the future use of miRNAs in diagnostics and therapeutics to improve pigs’ growth.

## 2. Materials and Methods

### 2.1. Animals and Sample Collection

Animal care and tissue collection procedures were approved by the Animal Care Committee of the Leibniz Institute for Farm Animal Biology and carried out in accordance with the approved guidelines for safeguarding good scientific practice at the institutions in the Leibniz Association and the measures were taken to minimize pain and discomfort and accord with the guidelines laid down by the European Communities Council Directive of 24 November 1986 (86/609/EEC). For this study, the animals were used for meat production and underwent no experimental treatment, diagnostic sampling, or any other intervention before killing therefore not requiring specific ethical approval. Animal handling as well as the killing was in accordance with applicable laws, relevant guidelines, and provisions for ethical regulations.

Analyses were done in a three-generation porcine F2 population, based on reciprocal crossbreeding DL and Pi breeds. One F1 father was mated to 11 F1 dams to produce a population of F2 fetuses. The sows were slaughtered, and the fetuses were extracted from the uteri and their weight was recorded. The discordant sib pair representing extremes for the fetuses’ weight was selected from the 118 F2 fetuses. Fetuses from eight families with significant differences in weight were categorized as intrauterine growth restricted (IUGR; *n* = 12) and appropriate for gestational age (AGA; *n* = 12) groups. Fetuses weighing more than two SD below the mean weight of their littermates were categorized as IUGR. From each family, either only female or only male or both discordant sib pairs were placed in the IUGR and AGA groups. Longissimus dorsi muscle tissue (LDM) from AGA and IUGR fetuses was collected and immediately frozen in liquid nitrogen and stored at −80 °C until RNA extraction.

### 2.2. RNA Isolation

Total RNA was isolated from LDM using the Tri-Reagent and RNeasy Mini kit (Qiagen) with an on-column DNase treatment according to the manufacturer’s protocol. The RNA integrity was assessed on a 1% agarose gel by electrophoresis. The RNA concentration was measured by a Nano Drop ND-1000 Spectrophotometer (PEQLAB). The quality and quantity of small RNA were assessed with an Agilent 2100 Bioanalyzer (Agilent) using an Agilent small RNA kit.

### 2.3. Gene Expression Profiling

The Porcine Snowball Microarray (Affymetrix) containing 47,880 probe sets was used to determine the expression profile of the LDM from F2 pigs at 63 dpc. Using the Affymetrix WT plus Expression kit and Genechip WT terminal labeling and hybridization kit according to the manufacturer’s instructions, 500 ng total RNA isolated from each tissue sample was used for cDNA synthesis and subsequent biotin labeling. Each of the labeled cRNA samples was hybridized on the array. The hybridization, washing, and scanning of the arrays were performed in accordance with the manufacturer’s recommendations. Affymetrix GCOC1.1.1 software was used for quality control. Expression Console software was used for robust multichip average (RMA) normalization and the detection of present genes by applying the detection above background (DABG) algorithm. Further filtering was done by excluding transcripts with low signals and probe sets that were present in less than 80% of the samples. For further analyses, 11,288 probe sets passed the quality filtering and were used. The expression data are available in the Gene Expression Omnibus public repository with the GEO accession number GSE169092.

### 2.4. MicroRNA Microarray Analysis

Affymetrix customized microarrays (GEO: GPL14969) were used [42]. Targets for hybridization were prepared from miRNA with the FlashTag™ Biotin RNA Labeling Kit for Affymetrix GeneChip miRNA arrays (Genisphere, Hatfield, PA, USA) according to the manufacturer’s recommendations. Briefly, 250 ng of miRNA of each individual was poly(A)-tailed using ATP–poly-A-Polymerase, then FlashTag Biotin end-labelled. After the hybridisation of biotin-labelled complementary RNA, chips were washed and processed to detect biotin-containing transcripts by Streptavidin-PE (Phycoerythrin) conjugate, then were scanned on GeneChip scanner 3000 7G (Affymetrix, Santa Clara, USA). Data were extracted from the images, and spots were quantified and processed by quality filtering. Expression Console software was used for robust multichip average (RMA) normalization and the detection of present miRNAs by applying the DABG (detection above background) algorithm. Further filtering was done by excluding probe sets that were present in less than 80% of the samples and annotated miRNAs that had a sequence greater than or equal to 30 nucleotides in length. For further analysis, 675 probe sets passed the quality filtering and were used. The expression data are available in the Gene Expression Omnibus public repository with the GEO accession number GSE169093.

### 2.5. Differentially Expressed Genes

To determine whether there were differences in the gene expression based on fetal weight groups, the normalization expression data were served as dependent variables as input for variance analysis using JMP Genomics 9.0 (SAS Institute, Cary, NC, USA). Fetal weight groups and sex were regarded as fixed effects and dam as a random effect. The adjusting for multiple comparisons across the Type 3 tests for all of the effects was calculated using the post hoc Tukey–Kramer test. No miRNA reached 5% FDR when comparing fetuses groups of IUGR and AGA. Therefore, we considered *p* < 0.05 as a significant threshold. For mRNA data, *p* values were adjusted according to the Benjamini–Hochberg method to control for FDR [43]. Genes with FDR-adjusted *p* values < 0.05 were considered as differentially expressed.

### 2.6. Prediction of miRNA Target Genes and Their Correlation Analysis

To investigate the downstream target mRNAs for differentially expressed miRNAs between IUGR and AGA fetuses, 17,065 3’-UTR sequences, 16,857 5’-UTR sequences, and 20,310 coding sequences were extracted from the *Sus scrofa* genome (Sscrofa11.1) based on Ensembl annotation version 102. These sequences were fragmented into 2000 base pair fragments with a 50-base overlap. Using the whole mature miRNA sequence, RNAhybrid version 2.1.2 was used to predict the target genes of differentially expressed miRNAs by setting the parameter as for a single hit per target, human-based assumed *p*-value distribution, minimum free energy (MFE) threshold of <−25 kcal/mole, and helix constraint from base 2 to 7 [44,45]. The Pearson correlation between miRNAs and mRNAs was calculated. Only negatively correlating miRNA–mRNA pairs were used for further analyses.

### 2.7. Correlation Network Analysis of miRNAs and Their mRNA Targets

To identify the miRNAs previously linked with IUGR or skeletal muscle growth, a thorough PubMed search was performed using the easyPubmed package (version 2.13) [46] in the R programming environment. Ten upregulated miRNAs were identified as novel miRNAs with no previously reported association with IUGR or skeletal muscle growth. Using their negatively correlated downregulated target genes with correlation coefficient ≤ −0.3, the correlation-based network was generated using the MetScape (version 3.1.3) plugin in the Cytoscape (version 3.6.1) environment [47].

### 2.8. Enrichment Analysis of miRNAs and Target mRNAs

Pairs of upregulated miRNAs were made with their downregulated target mRNAs, which are negatively correlated to each other (correlation coefficient < −0.30). Similarly, pairs of downregulated miRNAs were made with their upregulated target mRNAs, which are negatively correlated to each other (correlation coefficient < −0.30). In the first step, miRNA–mRNA pairs from upregulated miRNAs and downregulated genes were used for downstream gene ontology analysis for biological processes and KEGG pathways enrichment analysis using the ClueGO (version 2.5.1) and Cluepedia (version 1.5.7) plugin in Cytoscape (version.3.8.2) environment [48,49,50]. In the second step, miRNA–mRNA pairs from downregulated miRNAs and upregulated genes were used for over-representation analysis (ORA) for gene ontology for biological processes and KEGG pathways enrichment analysis using the WEB-based Gene Set Analysis Toolkit (WebGestalt) [51,52]. A hypergeometric test was used for enrichment analysis and Benjamini–Hochberg correction was used for multiple testing correction and the *Sus scrofa* genome assembly as a reference. The KEGG pathways and gene ontologies with FDR ≤ 0.05 were considered significantly enriched.

### 2.9. Validation of Sequencing Data by qPCR

To validate miRNA and mRNA results, transcripts of some differentially expressed miRNAs and mRNA were quantified in LDM from AGA and IUGR groups by qPCR using the Fluidigm BioMark HD System. Briefly, 250 ng of each RNA sample was reverse transcribed using the reverse transcription kit (Invitrogen) following the manufacturer’s protocol. For specific target amplification, 5 μL pre-amplification sample mixture for each cDNA was prepared by mixing 2.5 μL PreAmp Master Mix, 1.25  μL of cDNA, 1  μL PreAmp Master Mix, 0.5 μL Pooled Delta Gene Assay Mix (500 nM), and 0.75 μL water. These reactions were incubated at 95  °C for 10 min, followed by 10 cycles of 15 s at 95 °C and 4 min at 60  °C, and then infinite hold at 4  °C. After incubation, the samples were cleaned using the exonuclease I treatment method. Cleaned samples were diluted 10-fold using a DNA suspension buffer. Fluidigm quantitative measurement runs were carried out with 96.96 dynamic arrays (Fluidigm Corporation, CA, USA) according to the manufacturer’s instructions. The data were analyzed with real-time PCR analysis software in the BioMark HD instrument (Fluidigm Corporation, San Francisco, CA). Actin beta (*ACTB*) and glyceraldehyde-3-phosphate dehydrogenase (*GAPDH*) were used as housekeeping controls for mRNA quantification, and cel-miR-39-3p was used as the internal control for miRNA quantification, and calculations were based on the 2^−ΔCt^ method. Sixteen femtomole cel-miR-39-3p RNA spike-in, corresponding to 2 × 10^8^ copy number of transcripts, was added in each miRNA sample before reverse transcription. The miRNA and mRNA primer sequences are listed in Table S1.

## 3. Results

### 3.1. Phenotypic Trait and Differential Expression of miRNAs and mRNAs

**Figure 1:**
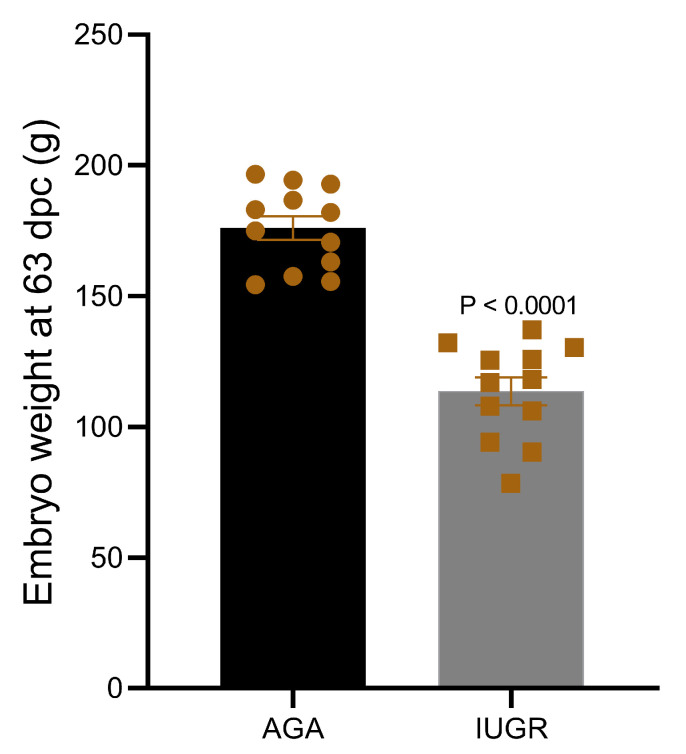
Weight of pig fetuses at 63 dpc in IUGR and AGA groups.

Discordant sibling pair design including eight different dams and one sire was used to group all fetuses based on their weight. Using the above described criteria, fetuses collected at 63 dpc were divided in two groups—intrauterine growth restricted fetuses (IUGR; *n* = 12) and appropriate for gestational age fetuses (AGA; *n* = 12). To minimize the effect of sex, an equal number of fetuses of the same sex from each dam were placed in both groups. Details of fetal phenotype from both groups are provided in Table 1. The mean weight of IUGR fetuses (113.60 ± 5.32 g) was significantly low compared to AGA fetuses (175.96 ± 4.5 g) (Figure 1).

**Figure 2:**
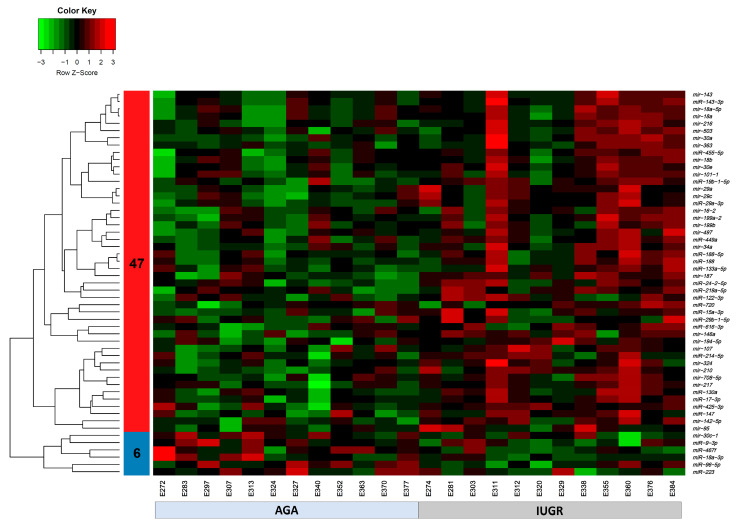
Heatmap of differentially expressed miRNAs (*p* < 0.05) in AGA and IUGR groups. Heatmap of miRNAs expression profiles was generated using the hierarchical clustering method of heatmap.2 function of gPlots (version 3.0.1) [53] Bioconductor Package in the R Programming environment (version 4.0.3). Forty-seven miRNAs in cluster 1 were upregulated and six miRNAs in cluster 2 were downregulated in LDM from IUGR compared to AGA fetuses. Upregulated miRNAs are marked in red, downregulated miRNAs are colored in green, and black color reflects no difference in expression levels.

**Figure 3:**
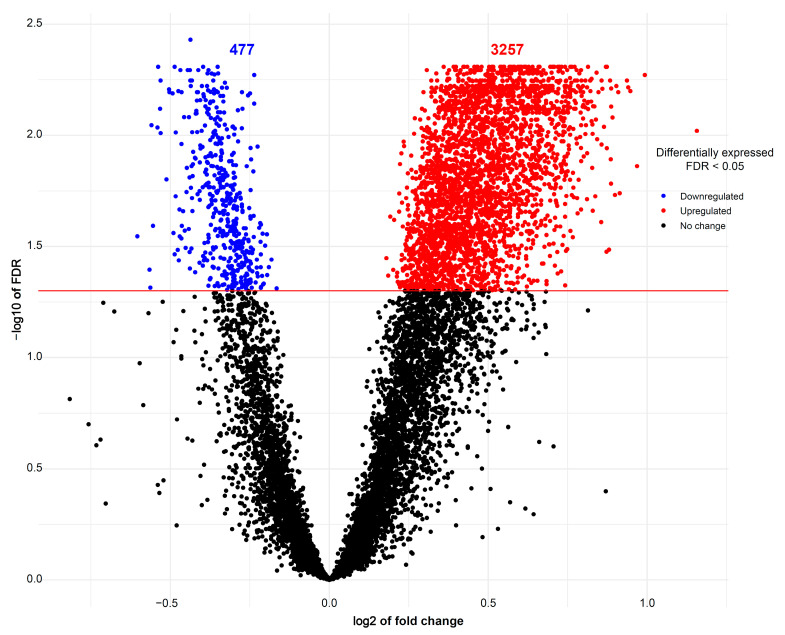
Volcano plots of mRNAs differentially expressed between AGA and IUGR groups. Red spots represent upregulated mRNAs and blue spots indicate downregulated mRNAs in LDM from IUGR fetuses compared to AGA fetuses. Black spots represent mRNAs that did not show changes between AGA and IUGR groups.

The expression of 675 miRNA probe sets was measured to investigate their association with the body weight of pig fetuses at 63 dpc. A total of 47 miRNAs were significantly upregulated (*p* < 0.05) and six miRNAs were significantly downregulated (*p* < 0.05) in longissimus dorsi muscle (LDM) from IUGR fetuses compared to AGA fetuses (Figure 2). The top five significantly different miRNA between groups were miR-210, miR-30, miR-17-3p, miR-199a, and miR-34a, which were all upregulated in IUGR fetuses. Moreover, out of 47,880 mRNA probe sets on the snowball microarray, 11,288 quality-filtered probe sets were further analyzed. A total of 477 genes were significantly downregulated (FDR < 0.05) and 3257 genes were significantly upregulated (FDR < 0.05) in LDM from IUGR fetuses compared to AGA fetuses (Figure 3).

### 3.2. MicroRNA–mRNA Networks

**Figure 4:**
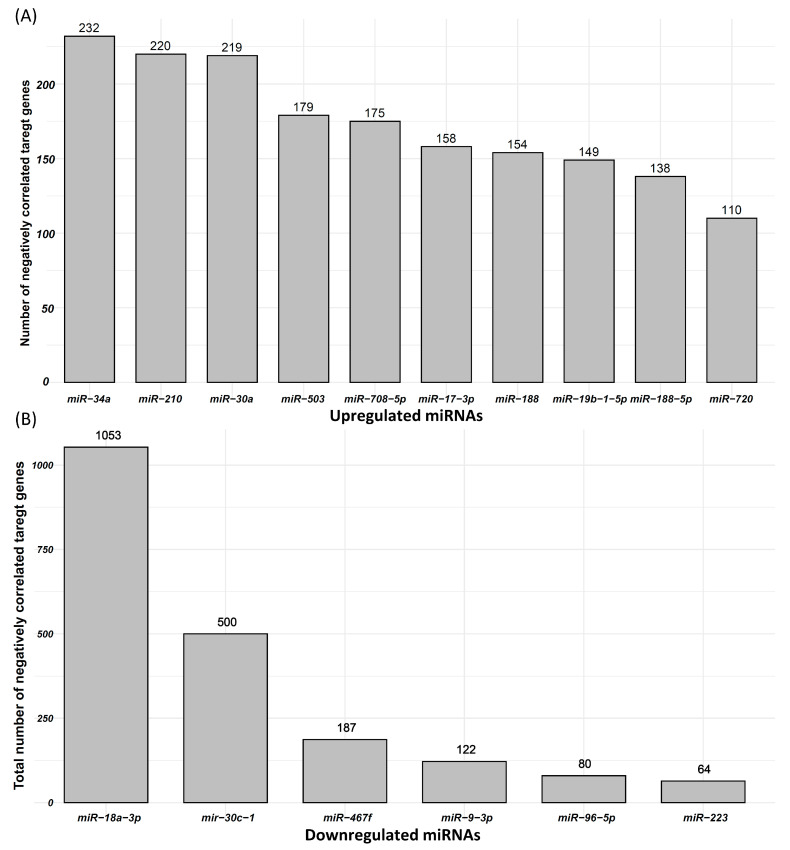
Differentially expressed miRNAs and their negatively correlated potential target genes. (**A**) Top 10 upregulated miRNAs and the number of their negatively correlated and downregulated target genes. (**B**) Downregulated miRNAs and the number of their negatively correlated and upregulated target genes.

Correlation between 47 upregulated miRNAs and 477 downregulated mRNAs in IUGR fetuses was calculated, and miRNA–mRNA pairs with correlation coefficient ≤ −0.3 were used for further analysis. Using RNAhybrid, we found potential target genes of 47 upregulated miRNAs in their negatively correlated downregulated genes. Our analysis showed that 47 upregulated miRNAs can potentially target 326 downregulated genes, which are also negatively correlated to these miRNAs. A total of 3292 possible miRNA–mRNA pairs that meet the described criteria are listed in Table S2. The correlation coefficient in these pairs ranges from −0.3 to −0.87 (Table S2). These miRNA–mRNA pairs were used for further downstream analysis. From these miRNA–mRNA pairs, we also analyzed a number of potential negatively correlated target genes of each upregulated miRNA. MiR-34, primarily known as a tumor-suppressing miRNA, was upregulated in IUGR fetuses and can target 232 downregulated genes, which are also negatively correlated to miR-34 (Figure 4A). Similarly, miR-210, a hypoxia responsive miRNA that has been frequently linked with IUGR in humans, was upregulated in IUGR fetuses and can target 220 downregulated genes, which are also negatively correlated to miR-210 (Figure 4A). The top 10 upregulated miRNAs and the total number of their potential targets among their negatively correlated downregulated genes are provided in Figure 4A.

We also measured the correlation between six downregulated miRNAs and 3257 upregulated mRNAs, and miRNA–mRNA pairs with correlation coefficient ≤ −0.3 were used in further analysis. Using RNAhybrid, we found target genes of six downregulated miRNAs in their negatively correlated upregulated genes. Our analysis showed that six downregulated miRNAs can target 1291 upregulated genes, which are also negatively correlated to these miRNAs. A total of 2007 possible miRNA–mRNA pairs that meet the described criteria are listed in Table S3. The correlation coefficient in these pairs ranges from −0.3 to −0.76 (Table S3). These miRNA–mRNA pairs were used for further downstream analysis. Six downregulated miRNAs including miR-18a-3p, miR-30c-1, miR-467f, miR-9-3p, miR-96-5p, and miR-223 can target 1053, 500, 187, 122, 80, and 64 upregulated genes, respectively (Figure 4B).

### 3.3. Gene Ontology and KEGG Pathway Enrichment Analysis of Downregulated Genes

**Figure 5:**
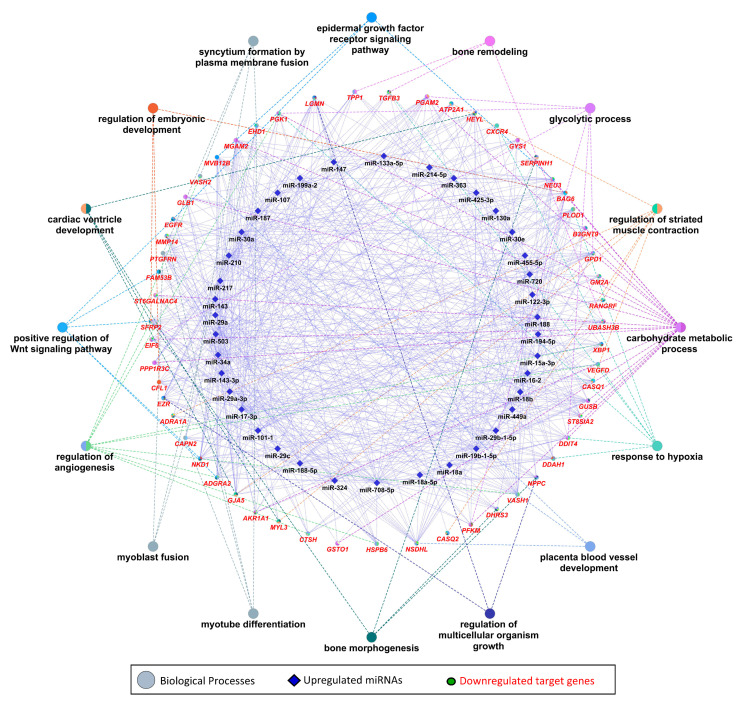
Enrichment analysis of downregulated genes, which are negatively correlated to and targeted by upregulated miRNAs, in gene ontologies for biological processes, derived using the ClueGO (version 2.5.1) and Cluepedia (version 1.5.7) plugin in Cytoscape (version 3.8.2) environment. Eclipses with red labels indicate the genes, and different pie colors in gene shapes indicate their enrichment in multiple biological processes. A complete list of biological processes enriched by downregulated genes is provided in Table S3.

**Figure 6:**
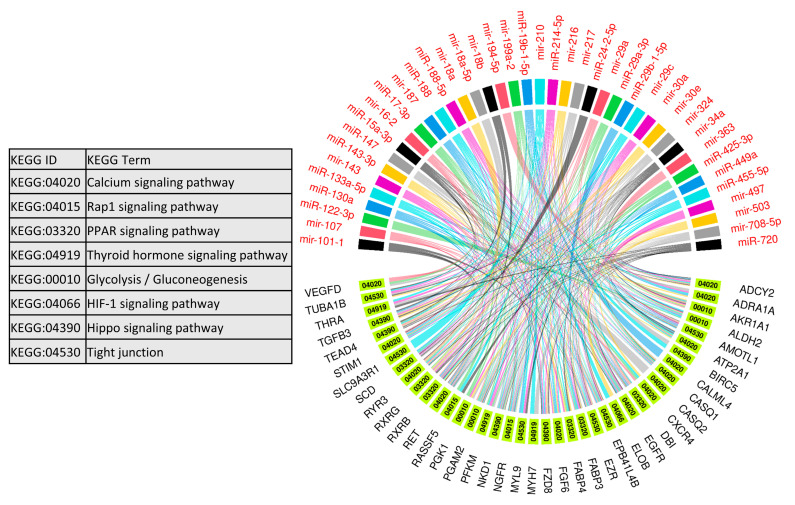
Enrichment analysis of downregulated genes, which are negatively correlated to and targeted by upregulated miRNAs, in KEGG pathways derived using the ClueGO (version 2.5.1) and Cluepedia (version 1.5.7) plugin in Cytoscape (version 3.8.2) environment. A complete list of KEGG pathways enriched by downregulated genes is provided in Table S3.

We used 47 upregulated miRNAs and their 326 downregulated target genes (Table S2) for gene ontology and KEGG pathway enrichment analysis using the ClueGO (version 2.5.1) and Cluepedia (version 1.5.7) plugin in Cytoscape (version.3.8.2) environment [48,49,50]. The results of gene ontology enrichment analysis showed that the downregulated genes significantly enriched biological processes vital for muscle growth and development, including myoblast fusion, myotube differentiation, and regulation of angiogenesis (Figure 5). The other important enriched biological processes included the regulation of embryonic development, bone remodeling, placental blood vessel development, regulation of multicellular organism growth, and epidermal growth factor receptor signaling pathway (Figure 5). Figure 5 also shows the upregulated miRNAs targeting the genes enriching important biological processes. A complete list of biological processes significantly enriched by downregulated genes is provided in Table S4. Similarly, KEGG pathways enrichment analysis showed that 326 downregulated genes significantly enriched important KEGG pathways including the calcium signaling pathway, Rap1 signaling pathways, PPAR signaling pathway, thyroid hormone signaling pathway, glycolysis/gluconeogenesis, HIF-1 signaling pathway, Hippo signaling pathways, and tight junction (Figure 6). Figure 6 also shows the upregulated miRNAs targeting the genes enriching important KEGG pathways. A complete list of significantly enriched KEGG pathways by downregulated genes is provided in Table S5.

### 3.4. Gene Ontology and KEGG Pathway Enrichment Analysis of Upregulated Genes

**Figure 7:**
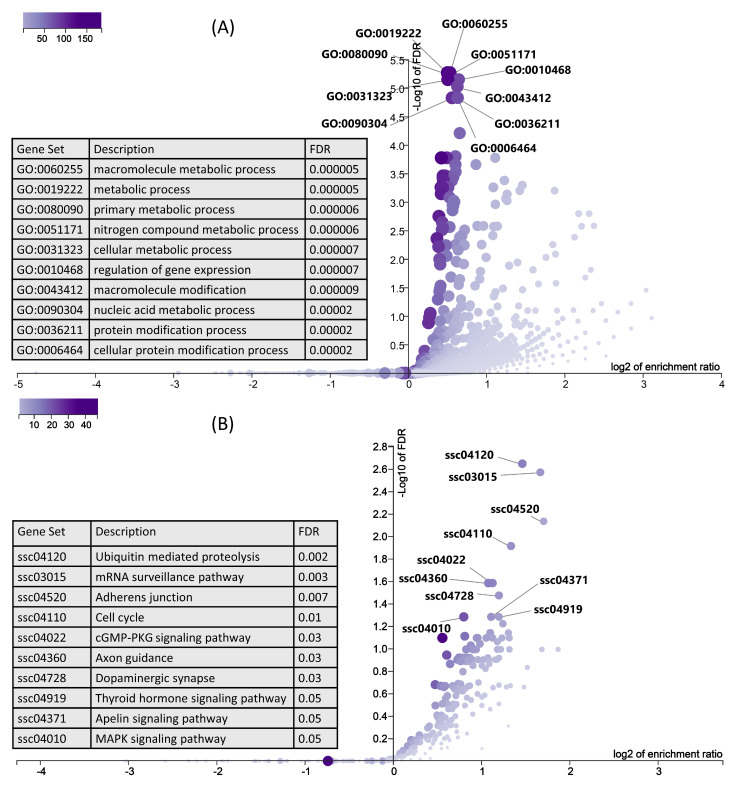
ORA results for upregulated genes, which are negatively correlated and targeted by downregulated miRNAs. (**A**) Biological processes significantly enriched by upregulated genes in LDM from IUGR fetuses at 63 dpc, derived using WebGestalt. A complete list of biological processes enriched by downregulated genes is provided in Table S4. (**B**) KEGG pathways enriched by upregulated genes in LDM from IUGR fetuses at 63 dpc, derived using WebGestalt.

In the next step, we used six downregulated genes and their 1291 upregulated target genes (Table S3) for gene ontology and KEGG pathway enrichment analysis using ORA in WebGestalt [51,52]. ORA results showed that the upregulated genes are enriched in important biological processes (Figure 7A). Interestingly, the top 10 enriched biological processes are mainly involved in metabolism, including macromolecule metabolism, primary metabolic process, cellular metabolic process, and nucleic acid metabolic process (Figure 7A). Similarly, KEGG pathways ORA analysis showed that the upregulated genes enriched important KEGG pathways including ubiquitin mediated proteolysis, the mRNA surveillance pathway, thyroid hormone signaling pathways, and the MAPK signaling pathway (Figure 7B).

### 3.5. Novel miRNA–mRNA Networks

**Figure 8:**
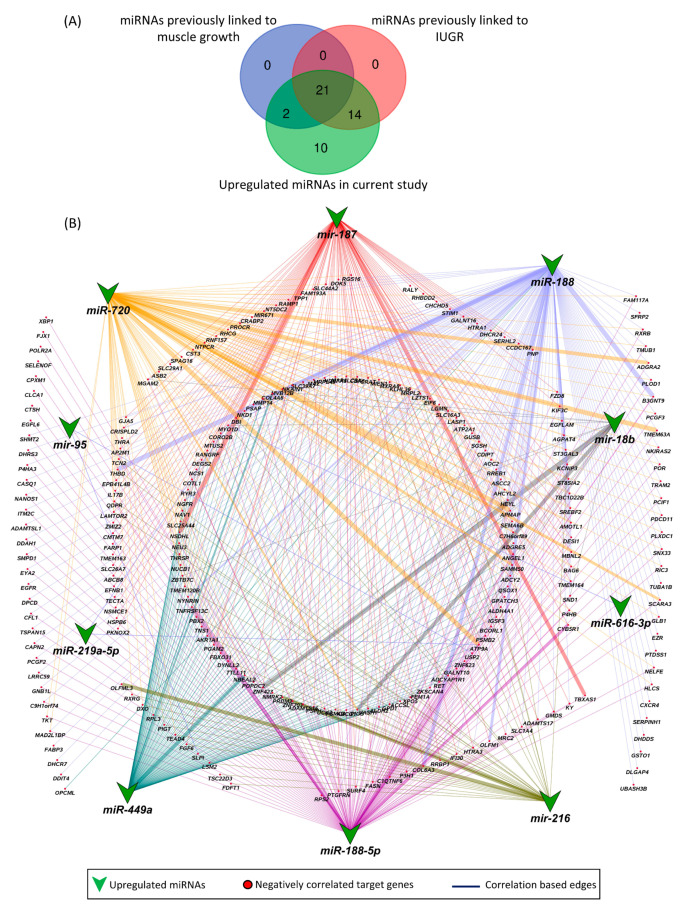
Novel miRNA–mRNA networks. (**A**) Comparison of upregulated miRNAs in LDM from IUGR fetuses with previously identified miRNAs associated with IUGR and skeletal muscle growth. (**B**) Correlation network between novel upregulated miRNAs and their negatively correlated downregulated target genes. A complete list of novel miRNA–mRNA pairs is provided in Table S5. Correlation coefficient in these pairs ranges from −0.3 to 0.74.

Other than previously known miRNAs, we also found 10 novel miRNAs that were upregulated in LDM of IUGR fetuses and have not been previously linked to either IUGR or skeletal muscle growth. These novel miRNAs included miR-187, miR-188, miR-188-5p, miR-18b, miR-216, miR-219a-5p, miR-449a, miR-616-3p, miR-720, and miR-95. Out of a total of 326 downregulated target genes, the upregulated novel miRNAs are negatively correlated and can target 253 downregulated genes (Figure 8). A complete list of the novel miRNA–mRNA pairs is provided in Table S6.

### 3.6. Validation by qPCR

**Figure 9:**
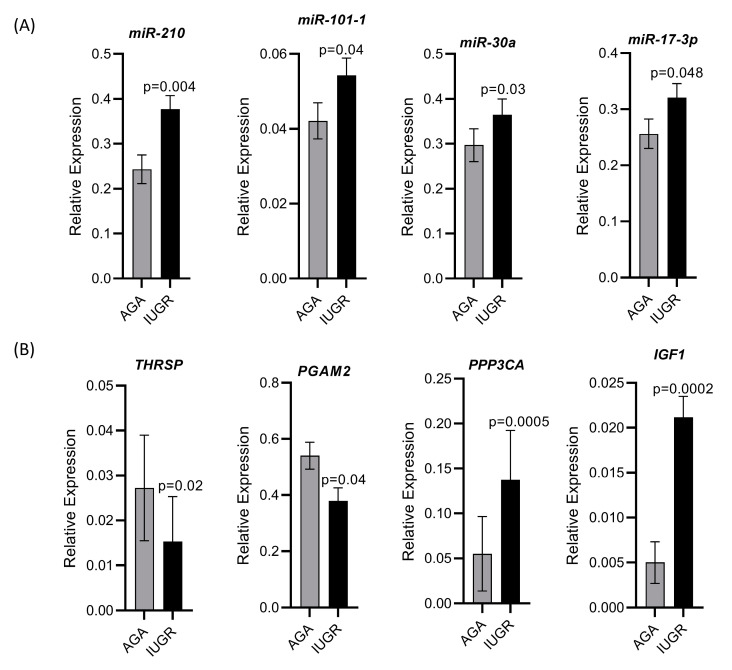
Validation of sequencing data by qPCR for selected miRNAs and mRNAs. (**A**) Relative expression of four upregulated miRNAs quantified through qPCR. (**B**) Relative expression of two downregulated and two upregulated mRNAs quantified through qPCR.

We validated the holistic expression analyses through qPCR for four upregulated miRNAs including miR-210, miR-101-1, miR-30a, and miR-17-3p, two downregulated genes including thyroid hormone responsive (*THRSP*) and phosphoglycerate mutase 2 (*PGAM2*), and two upregulated genes including protein phosphatase 3 catalytic subunit alpha (*PPP3CA*) and insulin like growth factor 1 (*IGF1*). All miRNAs and mRNAs quantified through qPCR showed the same trend of regulation as seen in the sequencing results (Figure 9).

## 4. Discussion

Normal prenatal skeletal muscle growth and development is a major determinant of the postnatal growth and survival and carcass quality of pigs [17,18,19]. The dynamic transition between different cell types during prenatal skeletal muscle development is regulated by numerous signaling pathways and transcription factors, and their dysregulation can lead to abnormal muscle development and reduced muscle mass [54]. We found significantly increased levels of 47 miRNAs in longissimus dorsi muscle (LDM) from IUGR fetuses compared to AGA fetuses at 63 dpc (Figure 2). In recent studies, we investigated the role of miRNAs in pathophysiology of pregnancy-associated disorders and identified potential biomarker miRNAs for pregnancy complications [55,56,57]. Out of 47 upregulated miRNAs reported in this study, dysregulation of five miRNAs including miR-34a, miR-210, miR-199a, miR-363, and miR-16 in placenta or maternal circulation has been previously linked with pathophysiology of IUGR [57,58]. According to our results, from downregulated genes in LDM from IUGR fetuses, miR-34a, miR-210, miR-199a, miR-363, and miR-16 can potentially target 232, 220, 99, 29, and 29 genes, respectively (Table S2). Previous evidence of dysregulation of miR-34a, miR-210, miR-199a, miR-363, and miR-16 in placenta or maternal circulation in IUGR pregnancies, and upregulation of these miRNA in skeletal muscle of IUGR fetuses in our study, indicates the diagnostic and therapeutic importance of these miRNAs.

The wide range of genes targeted by miR-34a and miR-210 makes them more interesting candidate miRNAs that are critical in the pathophysiology of IUGR. Previous studies have shown that miR-34a regulates skeletal muscle ageing [59], and its circulating levels in the body increase with age [60]. Myostatin (*MSTN*), a well-studied inhibitor of skeletal muscle growth and development, operates through miR-34a, and C2C12 cells treated with MSTN show an increase in miR-34a level [61]. Therefore, increased miR-34a in prenatal skeletal muscles can negatively regulate muscle growth leading to IUGR. Out of 326 downregulated genes in LDM from IUGR fetuses in this study, miR-34a can potentially target 232 genes, which indicates the involvement of miR-34a in a wide range of molecular pathways. Induced under hypoxia, miR-210 is one of the most studied miRNAs [62]. During skeletal muscle development, miR-210 is induced upon myogenic differentiation and its activation is regulated by hypoxia-inducible factor 1-α (*HIF1A*) [63]. It also regulates energy balance, mitochondrial function, and apoptosis in skeletal muscle cells [64]. According to our data, out of 326 downregulated genes in LDM from IUGR fetuses, miR-210 can potentially target 220 genes involved in critical biological processes for muscle growth and fetal development.

Collectively, 47 upregulated miRNAs are negatively correlated to and can target 326 downregulated genes in LDM from IUGR fetuses. Downstream enrichment analysis shows that the downregulated genes are involved in several biological processes and pathways critical for skeletal muscle development and fetal growth, such as myoblast fusion, angiogenesis, embryonic development, and bone morphogenesis (Figure 5). Myoblast fusion is a critical process during prenatal skeletal muscle development, as well as during postnatal muscle regeneration [65,66]. Plasma membrane fusion of mononucleated myoblasts with each other or with previously formed syncytial myofibers is the primary mechanism of increasing muscle mass [67]. In current study, among the downregulated genes in skeletal muscles from IUGR fetuses, calpain 2 (*CAPN2*), EH-domain containing 1 (*EHD1*), prostaglandin F2 receptor negative regulator (*PTGFRN*), and vasohibin-2 (*VASH2*) are involved in myoblast fusion (Figure 5). These genes were also identified as potential targets of upregulated miRNAs. *CAPN2* can be targeted by miR-188, miR-188-5p, miR-19b-1-5p, miR-29b-1-5p, miR-30a, miR-503, and miR-708-5p; *EHD1* is a potential target of miR-17-3p, miR-210, miR-34a, miR-425-3p, and miR-708-5p; and *PTGFRN* can be potentially targeted by miR-133a-5p, miR-15a-3p, miR-17-3p, miR-187, miR-188, miR-188-5p, miR-19b-1-5p, miR-30a, miR-30e, miR-324, miR-34a, miR-503, and miR-708-5p; whereas *VASH2* is a potential target of miR-210. Three out of these four genes are regulated by miR-34a and/or miR-210. We suggest that dysregulation of the genes involved in myoblast fusion during embryonic stages can impair skeletal muscle development and lead to IUGR.

Skeletal muscle is a highly vascularized tissue, and a microvasculature unit in skeletal muscle is comprised of three to four adjacent myofibers and five to 10 capillaries [68]. Vascularization of skeletal muscle is critical for its proper growth, development, and regeneration [69]. Our results show that six downregulated genes including cathepsin H (*CTSH*), heat shock protein beta-6 (*HSPB6*), secreted frizzled related protein 2 (*SFRP2*), *VASH2*, vascular endothelial growth factor D (*VEGFD*), and X-box binding protein 1 (*XBP1*) are associated with positive regulation of angiogenesis (Table S4). These six downregulated genes are targeted by different upregulated miRNAs. *CTSH* can be targeted by miR-15a-3p, miR-188-5p, mir-194-5p, miR-19b-1-5p, mir-217, miR-29b-1-5p, mir-34a, and mir-503; HSBP6 can be targeted by mir-187, mir-210, mir-29a, miR-29a-3p, mir-29c, mir-30a, mir-34a, and mir-708-5p; *SFRP2* is a potential target of miR-130a, miR-143-3p, miR-15a-3p, miR-17-3p, miR-188, miR-19b-1-5p, miR-210, miR-214-5p, miR-29a, miR-29a-3p, miR-29c, miR-34a, miR-455-5p, miR-503, and miR-708-5p; *VASH2* is targeted by miR-210; and *VEGFD* is targeted by miR-107, miR-16-2, miR-17-3p, and miR-324; whereas *XBP1* can be targeted by miR-15a-3p, miR-17-3p, miR-188-5p, miR-19b-1-5p, miR-210, miR-30a, miR-324, miR-34a, miR-425-3p, miR-503, and miR-708-5p (Figure 5, Table S2). Five out of six genes involved in positive regulation of angiogenesis are regulated by miR-210 and/or miR34a. We suggest that reduced expression of these genes in skeletal muscles during prenatal stages can not only affect prenatal skeletal muscle growth leading to IUGR, but can also have long-lasting postnatal affects.

Out of 47 upregulated miRNAs in LDM from IUGR fetuses, 37 miRNAs have been previously linked to either IUGR or skeletal muscle growth, 21 of which have been associated with both IUGR and skeletal muscle growth (Figure 8A, Table S6). Of these 21 miRNAs, dysregulation of miR-133a [70,71,72,73,74] and miR-29a [75,76] have been most often associated with IUGR and skeletal muscle growth. MiR-133a is one of the most studied muscle-specific miRNAs (myomiRs) [71], which promotes myoblast differentiation and reduces their proliferation [70]. According to our results, miR-133a is upregulated in LDM from IUGR fetuses and is negatively correlated and can potentially target 82 downregulated genes (Table S2). Our results further demonstrate that miR-133a target genes enriched biological processes critical for skeletal muscle growth and fetal development, including regulation of angiogenesis, placenta blood vessel development, myotube differentiation, and myoblast fusion (Figure 5). MiR-29a is a member of the miR-29 family of miRNAs which contains three members; miR-29a, miR-29b, and miR-29c [75]. By regulating the Wnt signaling pathway, miR-29a/b promotes osteogenesis [77,78]. Members of the miR-29 family target myogenin, serine/threonine kinase 3 (*AKT3*), and atrophy-related genes such as muscle ring-finger protein-1 (*MuRF1*), atrogin-1, and histone deacetylase 4 (*HDAC4I*) [75,79,80]. Our results show that miR-29a/b/c are significantly upregulated in LDM from IUGR fetuses and are negatively correlated and can potentially target 92 downregulated genes (Figure 2, Table S2). The miR-29 target genes enriched important biological pathways including bone morphogenesis, placenta blood vessel development, ADP metabolic process, and ATP generation from ADP. Placental insufficiency is one of the leading causes for the development of IUGR [6]. We suggest that dysregulation of miR-29 and their target genes can lead to serious impairments in skeletal muscle growth and placental development, contributing to the pathogenesis of IUGR.

Other than the regulation of key biological processes, downregulated target genes of upregulated miRNAs in LDM from IUGR fetuses also enriched important KEGG pathways including the calcium signaling pathway, thyroid hormone signaling pathway, glycolysis/gluconeogenesis, and Hippo signaling pathway (Figure 6). Calcium signaling plays a key role during different stages of prenatal muscle development as well as during postnatal maintenance and regeneration of skeletal muscles [81]. During the early developmental stages, calcium transients of long duration (80 s) occur only during a small time window during fetal development [81]. An artificial increase in the length of calcium transients negatively affects muscle development [82], indicating the importance of precise calcium signaling during skeletal muscle development. According to our results, 13 genes involved in calcium signaling are downregulated in LDM from IUGR fetuses and are potential targets of upregulated miRNAs. The hippo signaling pathway controls the development and size of multiple tissues and organs in the body, and is a critical regulator of skeletal muscle mass [83,84]. When active, the Hippo signaling pathway inhibits cell migration and proliferation by inhibiting proliferation-associated genes such as TEA domain transcription factor 4 (*TEAD4*), fibroblast growth factor (*FGF*), and baculoviral IAP repeat containing 5 (*BIRC5*) [85]. Interestingly, these genes are downregulated in LDM from IUGR fetuses, indicating increased activity of Hippo signaling pathways. Moreover, transforming growth factor beta (*TGFB*) targets the Hippo signaling pathway to stimulate cell invasion and proliferation [86]. Our data shows that *TGFB* is significantly reduced in LDM from IUGR fetuses, which can lead to the activation of the Hippo signaling pathway. We suggest that the increased activity of the Hippo signaling pathway in skeletal muscles from IUGR fetuses could be contributing to reduced muscle growth in these fetuses.

Pathophysiology of IUGR is not limited to only reduced prenatal fetal growth, but can also lead to long term problems after birth including insulin resistance, metabolic syndrome, and type 2 diabetes [87]. Hence, IUGR predisposes the fetus to development of postnatal or adult heath disorders [88,89], a phenomenon first introduced as Barker’s hypothesis [90]. Interestingly, the downregulated genes in LDM from IUGR fetuses enriched well-known metabolism-associated pathways including thyroid hormone signaling pathway, parathyroid hormone synthesis, secretion and action, and glycolysis/gluconeogenesis (Figure 6). In this context, the enrichment analysis of upregulated genes showed more interesting results. Upregulated target genes of downregulated miRNAs (Table S3) enriched important metabolic pathways. The majority of the top 10 biological processes enriched by upregulated genes are associated with metabolism, including the macromolecule metabolic process, primary metabolic process, nitrogen compound metabolic process, and nucleic acid metabolic process (Figure 7A). Moreover, upregulated genes in LDM from IUGR fetuses also enriched the thyroid hormone signaling pathway (Figure 7B). Thyroid hormones are critical for normal fetal growth and metabolism and facilitate the terminal differentiation of different fetal tissues [91]. They regulate important metabolic pathways such as glucose metabolism, lipolysis, and regulation of body weight [92]. Different studies have described the role of cellular metabolism in skeletal muscle development and regeneration [93,94,95]. Additionally, the most enriched pathway by upregulated genes is ubiquitin-mediated proteolysis (Figure 7B). Bachiller et al. recently reviewed ubiquitin-mediated proteolysis in several skeletal muscle disorders including muscle atrophy, defective growth of neuromuscular junction, nemaline myopathy, and reduced proliferation, and differentiation of myoblasts [96]. We suggest that dysregulation of metabolic pathways and upregulation of the ubiquitin pathway in skeletal muscles is not only associated with the pathophysiology of IUGR, but can also cause severe complications after birth and contribute toward increased morbidity and mortality in IUGR-affected piglets.

Due to their ability to target a wide range of genes and their involvement in the regulation of diverse biological processes, miRNAs have been under an immense focus to be used as diagnostic and therapeutic targets. Based on the existing evidence of trafficking of miRNAs through circulation and export of placental and fetal miRNAs to maternal circulation [97,98,99,100], we suggest that the dysregulated miRNAs in fetal skeletal muscles can be exported to other tissues and affect multiple organ systems in both the mother and the fetus. If detected in maternal circulation, the dysregulated miRNAs in fetal skeletal muscle can potentially serve as biomarkers of IUGR in pigs. However, further studies are needed to understand the roles of individual miRNAs in the pathogenesis of IUGR and to explore the possibility of the transport and identification of fetal skeletal muscle-originated miRNAs in maternal circulation.

## Figures and Tables

**Table 1:** Phenotype of fetuses in AGA and IUGR groups from one sire and eight dams.

AGA				IUGR			
Fetus ID	Fetal Weight (g)	Dam	Fetal Sex	Fetus ID	Fetal Weight (g)	Dam	Fetal Sex
E352	175	1	Male	E355	106.2	1	Male
E363	182	1	Male	E360	94.2	1	Male
E370	154.4	2	Female	E376	125.9	2	Female
E272	183	3	Female	E274	108	3	Male
E283	186.6	3	Male	E281	137.1	3	Female
E297	194.4	4	Male	E303	132.1	4	Male
E324	196.6	5	Male	E320	125.5	5	Male
E327	192.8	5	Male	E329	116.9	5	Male
E307	155.5	6	Female	E311	90.4	6	Female
E313	157.5	6	Male	E312	78.4	6	Male
E377	163.1	7	Female	E384	118.1	7	Female
E340	170.6	8	Female	E338	130.4	8	Female

## Data Availability

The expression data are available in the Gene Expression Omnibus public repository with the GEO accession number GSE169093.

## Supplementary Materials

The following are available online at https://www.mdpi.com/article/10.3390/cells10051007/s1: Table S1: qPCR primers, Table S2: Upregulated miRNAs and target genes, Table S3: Downregulated miRNAs and target genes, Table S4: BPs enriched by downregulated genes, Table S5: KEGG pathways enriched by downregulated genes, Table S6: Novel miRNA–mRNA networks
